# Supplementary material for: Content-rich biological network constructed by mining PubMed abstracts
Source: BMC Bioinformatics. 2004 Oct 8;5:147. doi: 10.1186/1471-2105-5-147 (PMC528731; doi:10.1186/1471-2105-5-147)
Supplement: Additional File 2 — The original results of the above study (non-essential files are deleted to keep the file size under the limit set by BMC bioinformatics). [file 1471-2105-5-147-S2.bz2 › chilibotAdditionalFile2/dip20/255ID7911228E1014/html/CDKN1A_PCNA.html]

 


 **CDKN1A** and **PCNA** 
  
Found 1649 abstracts in PubMed, retrieved 20.  
 

 What does Google say? 
 PDF only 
| .edu only 

---

**Interactive relationship** (e.g. stimulation, inhibition, etc)

**Inhibitory relationship**- Western blot analysis showed that both treatment with EGF alone and treatment with EGF after PH induce the expression of cyclins D1 and A and of p21 cip1  [ **CDKN1A** ] , but inhibites the expression of cyclin  [ **PCNA** ]  E and p27 kip1.  Ref: 12876382 Cell Physiol Biochem, 2003
- p21 Waf1  [ **CDKN1A** ]  Cip1  [ **CDKN1A** ]  exerts a key role in driving this G 2 exit both by inhibiting cyclin  [ **PCNA** ]  B1 Cdk1 and cyclin  [ **PCNA** ]  A Cdk1 2 complexes, which control G 2 M progression, and by blocking the phosphorylation of pRb family proteins.  Ref: 12881433 EMBO JEMBO J, 2003
**Neutral relationship**

**Non-interactive relationship** (e.g. studied together, co-existance, homology, etc.)

- AIMS To correlate the expression of a series of apoptotic and oncogene markers including p53, Bcl 2, BAX, Bcl XL, p21WAF, 1 CIP1  [ **CDKN1A** ] , cyclin  [ **PCNA** ]  D1, HER 2 neu in thymic epithelial tumours with histological type, stage and resectability and to determine whether the information on HER 2 neu would be valuable in identifying patients who are eligible for anti HER 2 neu treatment.  Ref: 12877732 Histopathology, 2003
- p XSC and p XSeSG were significantly more effective in the upregulation of pro apoptotic genes, such as p21 CIP1  [ **CDKN1A** ]  WAF1  [ **CDKN1A** ] , p27 KIP1, APO 1, and Caspase 3, while downregulating cell growth regulatory genes, such as c myc, cyclin  [ **PCNA** ]  D1, cyclin  [ **PCNA** ]  D2 and proliferating cell nuclear antigen  [ **PCNA** ]   **PCNA** .  Ref: 12844480 Carcinogenesis< MedlineTA>Carcinogenesis, 2003
- In our study 86 tonsil cancers were analysed for HPV status by sequence analysis of polymerase chain reaction products and for the expression of cell cycle proteins p53, p21 CIP1  [ **CDKN1A** ]  WAF1  [ **CDKN1A** ] , pRb, p16 INK4A, cyclin  [ **PCNA** ]  D1 and p27 KIP1 by immunohistochemistry.  Ref: 12845651 Int J Cancer, 2003
- Consistently with these data, Che 1 over expression inhibits proliferation of HCT116 and LoVo human colon carcinoma cell lines by activation of the cyclin  [ **PCNA** ]  dependent kinase inhibitor p21WAF1 Cip1  [ **CDKN1A** ]  in a p53 independent manner, and promoting growth arrest at the G1 phase of the cell cycle.  Ref: 12847090 J Biol Chem< MedlineTA>J Biol Chem, 2003
- These events were accompanied by down regulation of the Raf 1 MEK ERK pathway as well as diminished expression of Bcr Abl and cyclin  [ **PCNA** ]  D1, cleavage of p21 CIP1  [ **CDKN1A** ]  and pRb, and induction of the stress related kinases JNK and p38 MAPK.  Ref: 12893773 BloodBlood, 2003
- These effects were accompanied by increased association of 2 CDIs, p27 kip1 and p21 waf  [ **CDKN1A** ]  cip1  [ **CDKN1A** ] , with cyclin  [ **PCNA** ]  E Cdk2 complexes.  Ref: 12866041 Int J Cancer, 2003
- These deletions define a genomic region that includes cki 1 and cki 2, adjacent genes encoding members of the Cip  [ **CDKN1A** ]  Kip family of cyclin  [ **PCNA** ]  dependent kinase inhibitors.  Ref: 12885569 Dev Biol, 2003
- The cyclin  [ **PCNA** ]  dependent kinase inhibitor p21CIP WAF  [ **CDKN1A** ]  is a positive regulator of IGF 1 induced cell proliferation in MCF 7 human breast cancer cells.  Ref: 12867429 J Biol ChemJ Biol Chem, 2003
- Immunoblot analysis revealed that BMP2 treatment caused a down regulation of p53 family members and hence of cyclin  [ **PCNA** ]  dependent kinase inhibitor p21 WAF1  [ **CDKN1A** ] .  Ref: 12850001 Biochem Biophys Res Commun, 2003
- The aim of this study was to assess the effect of Fe chelators on p53 and the mechanism involved in the chelator mediated increase in mRNA levels of the universal cyclin  [ **PCNA** ]  dependent kinase inhibitor p21 CIP1  [ **CDKN1A** ]  WAF1  [ **CDKN1A** ]  Gao, J. and Richardson, D.R.  Ref: 12869419 CarcinogenesisCarcinogenesis, 2003
- Both 5% human fecal water and 4 micro mol L selenite greatly increased the mRNA level of the cyclin  [ **PCNA** ]  dependent kinase inhibitor gene p21 waf1  [ **CDKN1A** ] .  Ref: 12888658 J Nutr, 2003
- In 19 of these 63 patients, p53, p21 Waf1  [ **CDKN1A** ] , and proliferating cell nuclear antigen  [ **PCNA** ]   **PCNA**  were examined immunohistologically, and to quantify the risk of lymph node metastasis, computer analysis was performed on the basis of quantification theory II, in which pathological lymph node metastasis pN was the objective variable and high or low expression of each of the three markers was the predictive variable.  Ref: 12866574 J Exp Clin Cancer Res, 2003
- METHODS Immunohistochemical avdin biotin peroxidase complex ABC method was applied to 31 cases of fetal esophageal specimens and 194 cases of adult esophageal specimens to detect the expression of p53, p21 WAF 1  [ **CDKN1A** ]  and  **PCNA**  in fetal and adult esophageal epithelia.  Ref: 12854173 World J Gastroenterol, 2003
- Most thymomas and thymic carcinomas were negative for Bcl XL, p21WAF, 1 CIP1  [ **CDKN1A** ]  and cyclin  [ **PCNA** ]  D1.  Ref: 12877732 Histopathology, 2003
- Phagocytosis of serum and IgG opsonized zymosan SOZ and IOZ, respectively particles into J774A.1 macrophages induced apoptosis of the cells, accompanied by the expression of p21 WAF1  [ **CDKN1A** ] , one of cyclin  [ **PCNA** ]  dependent protein kinase CDK inhibitors.  Ref: 12858021 Exp Mol Med, 2003
- AIM To characterize the expression of p53, p21 WAF 1  [ **CDKN1A** ]  and proliferation cell nuclear antigen  **PCNA**  in fetal esophageal epithelia and to determine the role of these genes in proliferation of fetal and adult esophageal epithelial cells.  Ref: 12854173 World J Gastroenterol, 2003
- P21WAF1 Cip1  [ **CDKN1A** ] , an inhibitor of cyclin  [ **PCNA** ]  dependent kinases, is a critical downstream effector in the P53 specific pathway of growth control.  Ref: 12840864 J Tongji Med Univ, 1999
- Expressions of  **PCNA** , p53, p21 WAF 1  [ **CDKN1A** ]  and cell proliferation in fetal esophageal epithelia Comparative study with adult esophageal lesions from subjects at high incidence area for esophageal cancer in Henan, North China.  Ref: 12854173 World J Gastroenterol, 2003
- Prediction of lymph node metastasis by p53, p21 Waf1  [ **CDKN1A** ] , and  **PCNA**  expression in esophageal cancer patients.  Ref: 12866574 J Exp Clin Cancer Res, 2003
- These data demonstrate that the additive effect is likely predominantly due to the recruitment of p27 kip1 and, to a lesser extent, p21 waf  [ **CDKN1A** ]  cip1  [ **CDKN1A** ]  into the cyclin  [ **PCNA** ]  E Cdk2 complexes.  Ref: 12866041 Int J Cancer, 2003
